# Mechanical Properties and Acoustic Emission Characteristics of Anchored Structure Plane with Different JRC under Direct Shear Test

**DOI:** 10.3390/ma15124169

**Published:** 2022-06-12

**Authors:** Su Li, Hang Lin, Jingjing Feng, Rihong Cao, Huihua Hu

**Affiliations:** 1 School of Resources and Safety Engineering, Central South University, Changsha 410083, China; lisu1996@csu.edu.cn (S.L.); 195511018@csu.edu.cn (J.F.); 18229997417@163.com (R.C.); 2 Hunan Provincial Communications Planning, Survey and Design Institute, Changsha 410200, China

**Keywords:** anchored structural plane, direct shear test, JRC, *b*-value, RA-AF

## Abstract

Rock mass, the heterogeneous natural material composed of rock and discontinuities, is an important part of engineering construction. Discontinuities affect the mechanical properties of natural rock mass and further threaten the stability of rock engineering. To study the failure characteristics of anchored structure plane with different JRC, jointed specimens with four different JRC were fabricated by pouring cement mortar. Specimens were tested under four different normal loads to figure out how JRC and anchorage angle affect the mechanical properties of anchored structure plane. Besides, acoustic emission (AE) testing technology was adopted to explore the AE characteristics of anchored structural plane under shearing. The results showed that there exists a positive correlation between the peak shear strength and JRC. The undulation shape of structural plane led to an obvious downward trend in the strain softening stage of the structural plane with JRC of 6–8 and 18–20. When the anchorage angle ranged from 45° to 60°, the potentiation of bolt was the most significant. Based on the AE results, the larger the normal stress, the more likely the cumulative count curves were to enter the fast growth phase before the peak. The characteristics of *b*-value curves are mainly related to the topography of structural planes and whether the bolt is deformed. In the direct shear test, the cumulative proportion of shear cracks was more than 85%, which is much higher than that of tensile cracks. The variation of cumulative tensile cracks goes through three stages: slow growth, rapid growth, and slow growth. Compared with the unanchored structural plane, the variation range of real-time tensile cracks of the anchored structural plane is large, and sometimes the proportion of real-time tensile cracks may reach 80%.

## 1. Introduction

With the development of urbanization, higher requirements are put forward for resource exploitation and transportation, and various rock engineering such as mine slope, tunnels, and underground space engineering have been developed. As an important part of this rock engineering, it is particularly important to study the mechanical response of rock materials under different stress environments [1,2]. After a long period of geologic processes, the interior of the rock is destroyed and a number of discontinuities are formed. The existence of discontinuities weakens the strength of the rock and plays a crucial role in its failure process [3,4,5]. Many scholars have carried out a lot of research on structural planes and achieved some results [6,7,8,9]. The variation of rock joint morphology is complex and irregular [10,11]. To better describe the morphological characteristics of joints, Barton and Choubey [12] proposed 10 standard sections, which match well with rock joints in nature. The section length is 10 cm, and the specified roughness coefficient is 0–20. On the basis of JRC, many scholars have conducted in-depth research on the failure mechanism, strength characteristics, and related factors of structural plane through different methods [13,14,15,16].

Considering the structural plane plays an important role in the stability of engineering rock mass, a series of measures are used to reinforce engineering rock mass. Bolts can effectively limit the relative displacement between rock blocks and improve the overall stability of fractured rock mass. Furthermore, it possesses the advantages of simple technology, being safe, reliable, economic, and efficient, and is widely adopted in reinforcement engineering in slopes, underground protection engineering, and tunnels. Its good reinforcement effect has been unanimously recognized. At present, experts have systematically studied the anchored structural plane through theoretical derivation [17,18], laboratory experiment [19,20], and numerical simulation [21,22], and achieved abundant research results. Through laboratory tests, as the most common means to study the anchored structural plane, many scholars have carried out relevant research around rock strength [23], anchoring method [24], bolt type [25], anchorage angle [26], and joint roughness [27], and revealed the influence law of these factors on the shear mechanical properties of anchored structural plane [28,29]. According to some research [30], the irregular joint morphology is the main reason for the appearance of dilatancy. The normal displacement of rock block caused by the dilatancy will change the force condition of the bolt and then affect the shear mechanical properties of anchored structural plane. Yoshinaka et al. [31] carried out a direct shear test on a regular jagged anchored joint, and found that a larger undulating angle can make the bolt work better. Wang et al. [32] and Chen et al. [33] fabricated anchored jointed specimens with different roughness and carried out a direct shear test. They pointed out that the existence of the bolt enhances the deformability of rock mass, and the joint roughness will affect the deformation of the bolt.

Up until now, the experimental research on anchored structural plane has mainly focused on the shear strength of structural plane, and the research on the failure process and real-time microcrack failure characteristics is scarcer still [34]. As a means of monitoring rock deformation, AE technology can collect and record the transient elastic waves generated by rock under loading in real time. Thus, this study customized resin plates with different JRC information through 3D printing, fabricated anchored and unanchored specimens with different JRC, carried out direct shear experiments, and analyzed the effects of JRC, normal stress, and anchorage angle on mechanical characteristics. In addition, with the help of AE monitoring technology, the relationship between micro-failure characteristics of structural planes under direct shear and shear stress curves was studied, and the influence of JRC and anchorage angle on microscopic fracture characteristics of rough structural planes under direct shear test were analyzed. The variation of AE-derived parameters (*b*-value and RA-AF) can provide a reference for stability evaluation of engineering rock mass.

## 2. Experiment

Many scholars have widely used various rock-like materials [35,36,37] to develop experimental research, and their findings have been recognized. In order to study the mechanical behavior of the anchored structural plane under direct shear, cement mortar was adopted to fabricate specimens with structural plane of different JRC. The mass ratio of cement mortar is cement:sand:water = 1.5:0.8:0.6. Ninety mm of full thread rod with the material of 304 steel was selected to simulate the bolt, whose yield strength σ_y_ = 205 MPa and elastic modulus E = 199 GPa.

**Figure 1:**
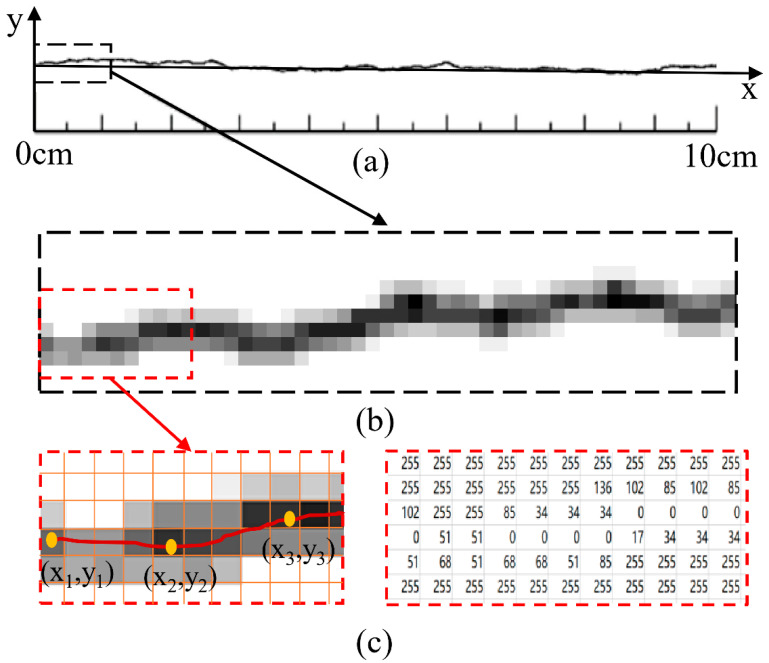
Extraction process of JRC profile coordinates. (**a**) standard JRC profile with value of 12-14 (**b**) basic gray image (**c**) gray matrix and intensity matrix.

In the current study [38,39], the specimen with structural plane was made by pouring on one side of the structural plane first, and then pouring on the other half after solidification. To make specimens with more realistic structural planes, in this paper, the roughness contour was digitalized by the gray image-processing method. In shooting and calculation, gray digital images will carry a lot of information. Each pixel in the image constitutes a sample. These kinds of images, called black-and-white images, consist only of gray shadows, taking Barton’s standard JRC profile with the value of 12–14 as an example (Figure 1a). Figure 1b shows the basic gray image of the 1 cm of this JRC curve. The built-in function of MATLAB can divide the gray image into a pixel grid as shown in Figure 1c. The part shown contains 160 pixels, with a width of 11 pixels and a height of 6 pixels. Each pixel can be represented by a value from 0 to 255. The grayer the pixel, the lower the gray value.

Comparing the intensity matrix with the gray image, it can be seen that the element of low intensity value is correlated with the height of the center line of the profile curve. Grayscale reflects the ordinate of each pixel of the contour, and the intensity shows how close each pixel is to the center line of the contour. The abscissa of these points is calculated by the relationship between the column number of gray matrix in digital image and the length of the contour. The conversion ratio c is expressed as:

(1) $$c=\frac{L}{M-1}$$

In Equation (1), *L* represents the actual length of the JRC contour image, and *M* represents the column number of gray matrix in digital image, which is equal to the number of pixels in the length direction of the JRC contour image. Taking Barton’s standard JRC profile with value of 12–14 as an example, the pixel grid of the image was 44 × 630, and the horizontal length of the standard JRC profile was 100 mm. Therefore, the conversion ratio was 0.158. Generally, the left endpoint is set as the origin, and then the abscissa $\left(x_{u}\right)$ of the pixel in the U-th column of the contour line can be expressed as:

(2) $$x_{u}=c\left(u-1\right)$$

Setting the distance between adjacent points to 1, the column position of the *M*-th pixel in the original gray matrix can be calculated as:

(3) $$u=1+\frac{l\left(m-1\right)}{c}$$

As the gray value of each pixel represents the proximity to the contour center line, the ordinate of the points on the contour line can be calculated by the gray value of each column:

(4) $$y_{u}=c\left(\frac{\sum_{v=1}^{A}v\left(255-I\left(v,u\right)\right)}{\sum_{v=1}^{A}\left(255-I\left(v,u\right)\right)}-\frac{\sum_{v=1}^{A}v\left(255-I\left(v,1\right)\right)}{\sum_{u=1}^{A}\left(255-I\left(v,1\right)\right)}\right)$$

In Equation (4), *A* represents the row number of gray matrix. 

**Figure 2:**
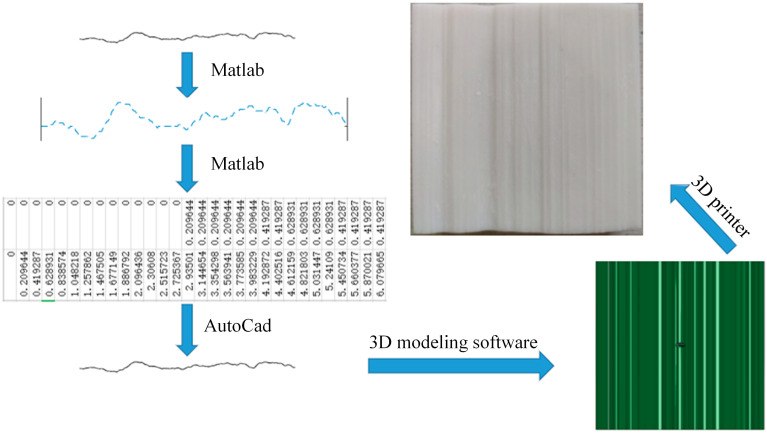
The realization process of resin plates recorded with the information of JRC.

According to the above principles, the coordinates (*x_u_, y_u_*) of points on the JRC profile could be output every 1 mm interval length through MATLAB, and a 3D model was drawn by 3D modeling software according to the extracted coordinate point data. Finally, a 3D printer was used to print it as resin plates recorded with different JRC (the center of the resin plate contains a prefabricated hole with a diameter of 3 mm for placing the bolt). The size of the resin plate was 100 mm × 100 mm × 30 mm. The realization process of the resin plate is shown in Figure 2

**Figure 3:**
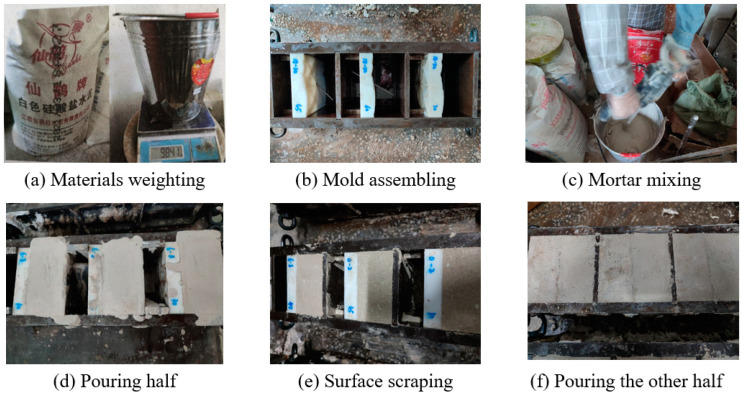
Fabrication of specimens with different JRC.

**Figure 4:**
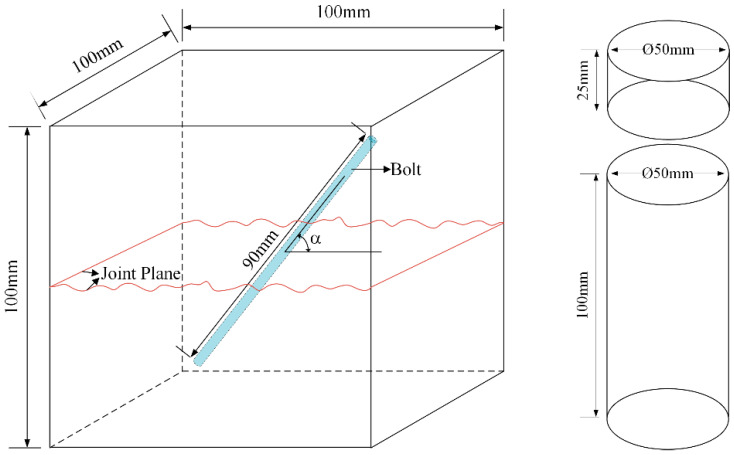
The diagrammatic sketch of specimens in this study.

The fabrication process of specimens with structural planes with different JRC is shown in Figure 3. When making unanchored specimens, the resin plate was first placed in the middle of the steel mold, and an appropriate pad was placed on the back of the resin plate to prevent it from deviating when pouring cement mortar. After that, the cement mortar was poured into one side of the mold. After 24 h, we removed the resin plate, brushed a layer of oil on the structural plane, and poured cement mortar into the other half of the steel mold, and then demolded after 1 day. All specimens were cured at constant temperature and humidity for 28 days. The manufacturing and curing process of anchored specimens is completely consistent with that of unanchored specimens except that the bolt needs to be fixed in the resin plate first. In this paper, the anchorage angle α was defined as the angle between the bolt and shear direction. α was 30°, 45°, 60°, 90°, and the number of bolts was 1, which was arranged in the center of the structural plane. The size of the specimen was 100 mm × 100 mm × 100 mm, and the geometric diagram of specimen is shown in Figure 4. Each specimen was assigned an ID, DS-ab-α-σ, where DS represents direct shear experiment, ab represents the range of JRC, α means the anchorage angle, and σ means the normal stress, which were 2 MPa, 3 MPa, 4 MPa, and 5 MPa respectively. According to the recommendation of ISRM [40,41], a batch of 50 mm × 50 mm and 50 mm × 100 mm standard specimens were fabricated.

**Figure 5:**
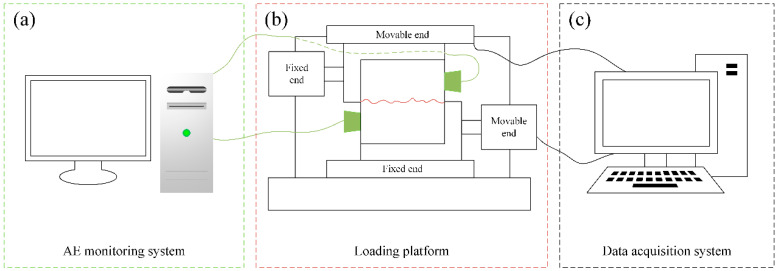
The arrangement of experimental devices. (**a**) AE monitoring system (**b**) Loading platform (**c**) Data acquisition system.

The YZW50 multifunctional rock direct shear apparatus was adopted in this study. The maximum of normal load and tangential load is 500 kN. The instrument can provide two loading methods, displacement control and stress control, which meet the requirements of the test. During the loading, the tangential and normal stress and displacement of the specimen were recorded automatically by the data acquisition system. The normal stress was first applied to the set-point (2 MPa, 3 MPa, 4 MPa, and 5 MPa), and then the tangential load was applied to the specimen until the final failure. The loading rate was 0.01 mm/min. The PCI-II AE monitoring system was adopted in the AE test. The relevant arrangement of facilities is shown in Figure 5.

## 3. Mechanical Properties

Uniaxial compression test, Brazilian splitting test, and triaxial test were carried out on the standard cylindrical specimens. The basic mechanical parameters of rock-like materials used in this paper are shown in Table 1. 

**Figure 6:**
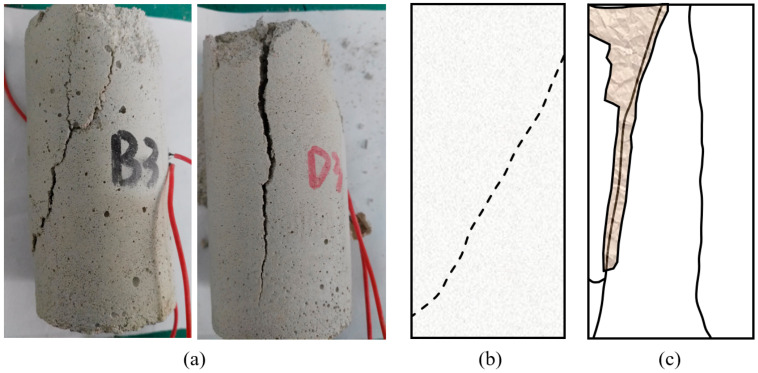
Final failure modes: (**a**) cement mortar, (**b**) sandstone-1 (**c**) sandstone-2.

As shown in Table 1, the unconfined compression strength was 34.13 MPa, the tensile strength was 2.98 MPa, and the brittleness index (compression–tension ratio) was 11.42. Besides, the elastic modulus of cement mortar is 5.27 GPa. These parameters are in good agreement with the mechanical properties of sandstone [42]. The failure modes of cement mortar and sandstone are shown in Figure 6. The typical failure modes of intact sandstone under uniaxial loading can be divided into slope-failure and split-failure [43]. It can be seen in Figure 6, the failure mode of cement mortar is consistent with that of sandstone [42,44], which means the rock-like material used in this paper can well simulate the natural rock.

**Figure 7:**
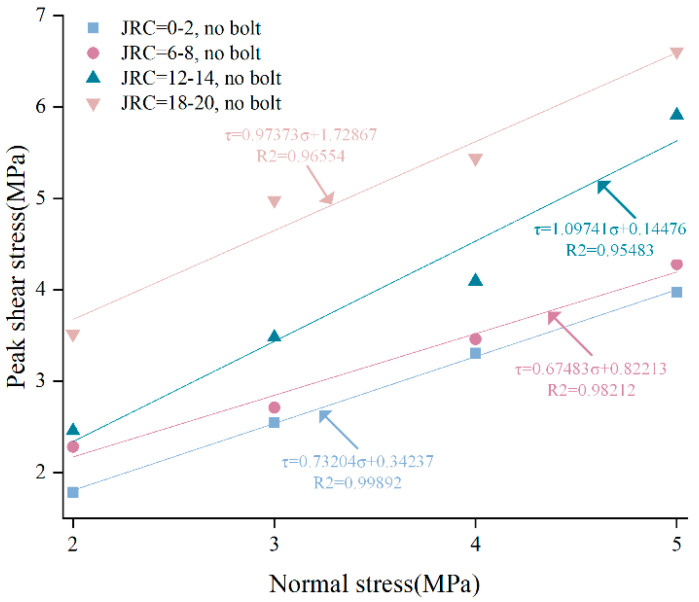
Fitting curves of shear strength parameters of unanchored structural planes with different JRC.

The peak shear strength of unanchored structural planes under different normal stresses were linearly fitted, and the relationship between peak shear stress of structural plane with different JRC and normal stress was obtained, as shown in Figure 7. It can be found that the regression coefficient R^2^ after linearly fitting were all greater than 0.95, indicating that the imitative effect is good and performs a reliable law. Cohesion-like stress and friction angle of the structural plane were calculated by using the Mohr–Coulomb criterion, as shown in Table 2. From Figure 7, under the same normal stress, the peak shear strength of unanchored structural plane increased with the increase of JRC, and the cohesion-like stress of unanchored structural planes with different JRC were 0.34 Mpa, 0.82 Mpa, 0.14 MPa, and 1.73 MPa, respectively. Based on the linear fitting parameters in Figure 7, friction angle of structural planes with different JRC could be inverted, which were 36.2°, 34.0°, 47.7°, and 44.2°, respectively. Except for the structural plane with JRC of 12–14, the larger the joint roughness, the larger the cohesion of unanchored structural plane. The roughness of the structural plane with JRC of 12–14 was large, but the cohesion-like stress was the smallest. This is because there existed a relatively large and smooth sawtooth on the structural plane with JRC of 12–14, and sliding is the main cause of specimen failure. The shear strength of the specimen is determined by cohesion and friction angle, which may explain why the cohesion-like stress of the structural plane with JRC of 12–14 was the minimum, but its friction angle was the maximum.

**Figure 8:**
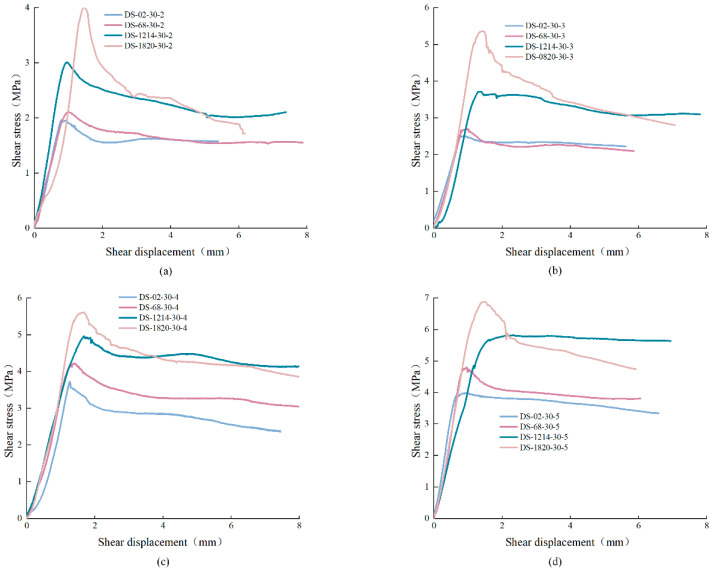
Shear stress-displacement curves of anchored structural planes under different normal stress: (**a**) σ = 2 MPa, (**b**) σ = 3 MPa, (**c**) σ = 4 MPa, (**d**) σ = 5 Mpa (specimen ID: DS-ab-α-σ, where DS represents direct shear experiment, ab represents the range of JRC, α means the anchorage angle, and σ means the normal stress).

**Figure 9:**
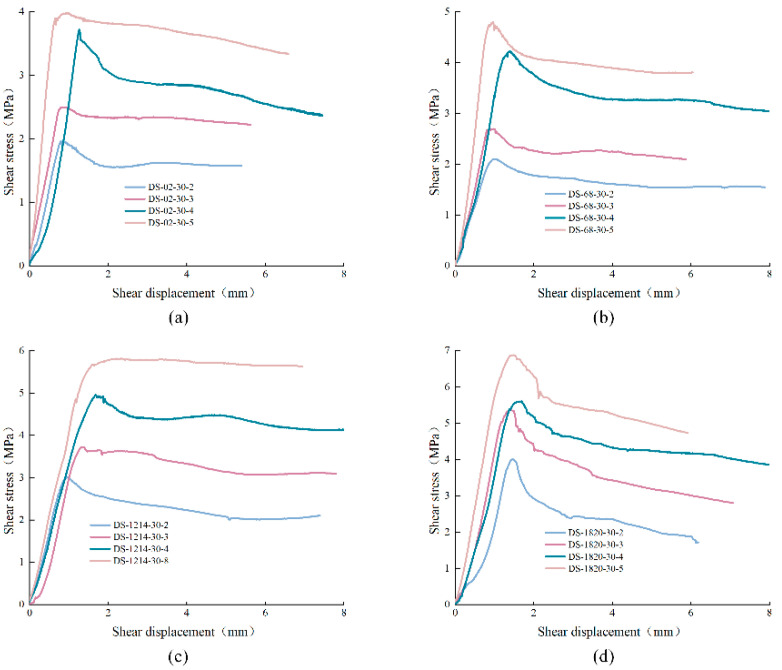
Shear stress-displacement curves of anchored structural planes with different JRC: (**a**) JRC = 0–2, (**b**) JRC = 6–8, (**c**) JRC = 12–14, (**d**) JRC = 18–20 (Specimen ID: DS-ab-α-σ, where DS represents direct shear experiment, ab represents the range of JRC, α means the anchorage angle, and σ means the normal stress).

The shear stress-displacement curves of anchored structural planes were classified and analyzed according to JRC and normal stress. Anchored structural planes with anchorage angle of 30° were taken as an example, as shown in Figure 8 and Figure 9. It is not difficult to find that the shear stress-displacement curves of anchored structural planes can be divided into four stages: elastic stage, plastic deformation stage, strain-softening stage, and residual stage respectively. In Figure 8, for specimens under the same normal stress, the peak shear strength increased with the increase of JRC, but the difference in shear strength of structural plane with JRC of 0–2 and 6–8 was small. The residual strength of anchored structural plane with JRC equal to 18–20 was less than that with JRC equal to 12–14. In addition, the characteristics of the strain-softening stage also differed greatly. When JRC was 0–2 and 12–14, the shear stress curves decreased less before entering the residual stage. When the normal stress was small, the shear stress-displacement curves of specimens with JRC of 12–14 did not show an obvious stress drop after the peak. For specimens with JRC of 6–8 and 18–20, the shear stress decreased greatly after the peak. This is mainly due to the fact that there are fewer jagged bulges in the structural planes with JRC of 0–2 and 12–14. Under shearing, the failure of the specimen is mainly caused by slip failure, which mainly overcomes the cohesive force. The structural planes with JRC of 6–8 and 18–20 had more serrated bulges. These bulges were cut off during the shear process, resulting in a large stress drop.

**Figure 10:**
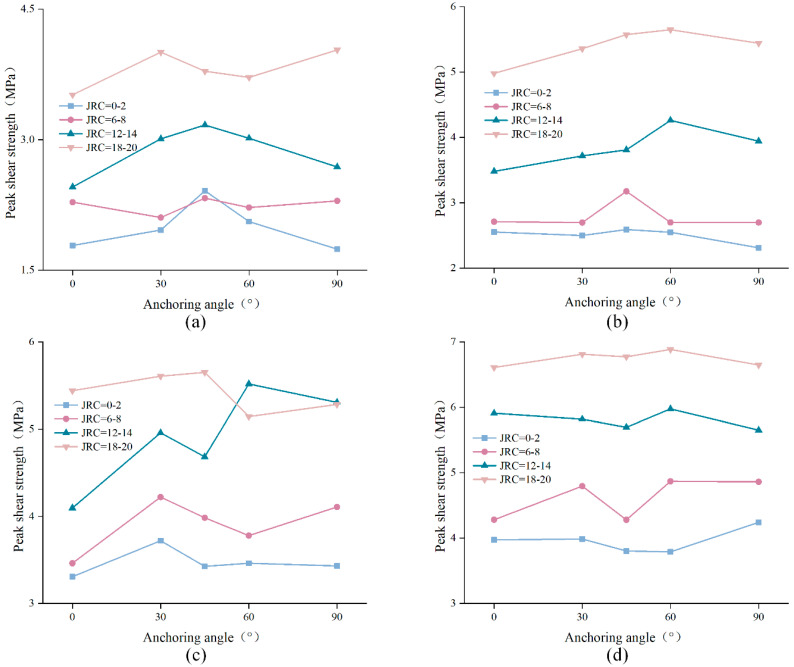
Relationship between shear strength and anchorage angle: (**a**) σ = 2 MPa, (**b**) σ = 3 MPa, (**c**) σ = 4 MPa, (**d**) σ = 5 Mpa.

The peak shear strength of the anchored structural plane was classified and drawn according to different normal stress and different JRC, as shown in Figure 10. It is easy to find that no matter how the JRC or normal stress changes, each curve roughly showed a change trend of rising first and then falling. Almost all curves reached the maximum when the anchorage angle was 45° or 60°, that is, the best installation angle of the bolt ranged from 45° to 60°.

## 4. AE Characteristics

Since the failure process of structural plane cannot be observed in real time during shearing, photographing and 3D morphology scanning can only compare and analyze the morphology characteristics before and after failure. In the process of rock deformation, a large number of cracks will be generated. With the initiation and propagation of these cracks, energy stored inside the rock is released in the form of elastic waves, which is called the rock AE phenomenon. Thus, AE signal characteristics can directly reflect the development degree of cracks in rock. As a good tool to study the failure evolution process of brittle materials, AE technology can continuously and real-time monitor the generation and propagation of micro-cracks in brittle materials under loading, which has been widely used to study the failure mechanism of rock, concrete, and other materials. During the experiment, the sensors were attached to the specimen. After receiving the AE signal, the weak mechanical vibration was transformed into electrical signal, which was amplified by the preamplifier, and then the mechanical noise was removed by the filter. The main amplifier further amplified the filtered signal for subsequent signal processing. AE parameters obtained from AE instrument are called direct AE parameters, including ring-down count, energy, peak frequency, amplitude and so on. Direct AE parameters can be processed to obtain derived AE parameters, such as *b*-value, RA-AF.

### 4.1. Ring-Down Count

Ring-down count is a direct AE parameter obtained from rock deformation by AE instrument. The ring-down count refers to the number of times the ringing pulse exceeds the threshold value in a unit time, and the total ring-down count in a certain period of time is called the cumulative ring-down count. Ring-down count can reflect the signal strength and frequency. The rapid rise of ring-down count means the intensification of failure degree. The failure process characteristics of the specimen can be seen from the variation trend of the cumulative ring-down count curve. The steeper the slope of the curve, the faster the crack generation speed, the flatter the slope of the curve, and the slower the crack generation speed. 

**Figure 11:**
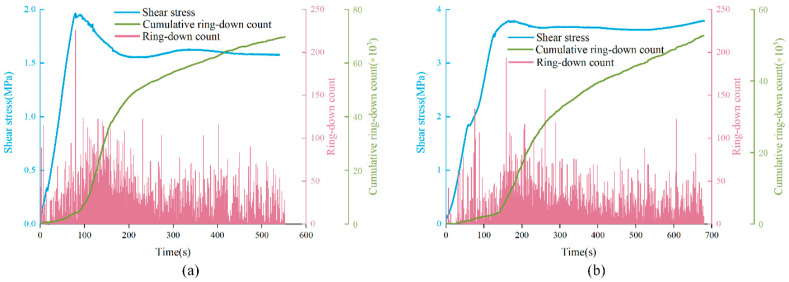
The variation trend of ring-down count and shear stress with time: (**a**) DS-02-30-2, (**b**) DS-02-60-5.

Figure 11 shows the characteristics of the ring-down count of anchored structural planes. It can be seen that the variation of ring-down count is consistent with the shear stress curve. The ring-down count increased in the pre-peak stage, and there was a sudden increase when the shear stress curve reached the peak value. In the strain softening stage, the ring-down count gradually decreased and tended to be flat in the residual stage. In the residual stage, the ring-down count remained at a certain intensity, around 60. In addition, the trend of cumulative ring-down count curve also has three stages, which rises slowly at the initial stage, then the rising rate becomes faster, and finally tends to slow down. For anchored specimens under low normal stress (2 MPa), in stage 1 (pre-peak stage), the cumulative ring-down curve rose slowly and AE signal was relatively weak, which means that there were few microcracks generated in this stage. In stage 2 (strain softening stage), the shear stress dropped in a relatively short time, and the rising rate of cumulative ring down count curve increased significantly, that is, the AE signal was strong. This means that the failure mainly occurred in stage 2. In stage 3 (residual stage), the rising trend of the curve gradually slowed down. For anchored specimens subjected to higher normal stress (greater than or equal to 3 MPa), although the trend of cumulative ring-down count curve was the same, it can be clearly found that the curve began to rise rapidly in stage 1, and then turned to slow growth in stage 3, which means that the structural plane started to fail in stage 1.

### 4.2. B-value

Due to the high similarity between AE phenomenon in the process of rock failure and seismic wave phenomenon, studying the variation law of *b*-value during the shear process can reveal the failure characteristics of rock. In the study of earthquake frequency and magnitude, Gutenberg and Richter [45] first proposed the *b*-value which are used to describe the distribution proportion of focal dimension and the famous G-R equation.

(5) lgN=a−bM

In Equation (5): *M* is magnitude and *N* is the number of the magnitude, which is greater than or equal to *M*.

Because of the similarity between acoustic emission and seismic wave, Equation (2) is often used in the study of acoustic emission of rock failure. The unit of amplitude of AE is decibel (dB). Therefore, the M value in Equation (2) can be replaced by the amplitude divided by 20 in calculation. The equation that can be used for the rock is as follows:

(6) $$\mathrm{lg}N=a-b(A_{dB}/20)$$

In Equation (6), *N* is the frequency increment of AE amplitude greater than or equal to the threshold value, *A_dB_* is the amplitude in dB, a is a constant, and *b* represents *b*-value. *b*-value can indicate the degree of fracture in the rock mass. The increase of *b*-value means that the rock fracture is mainly caused by small cracks, and the decrease of *b*-value represents that the rock fracture is mainly caused by large cracks.

**Figure 12:**
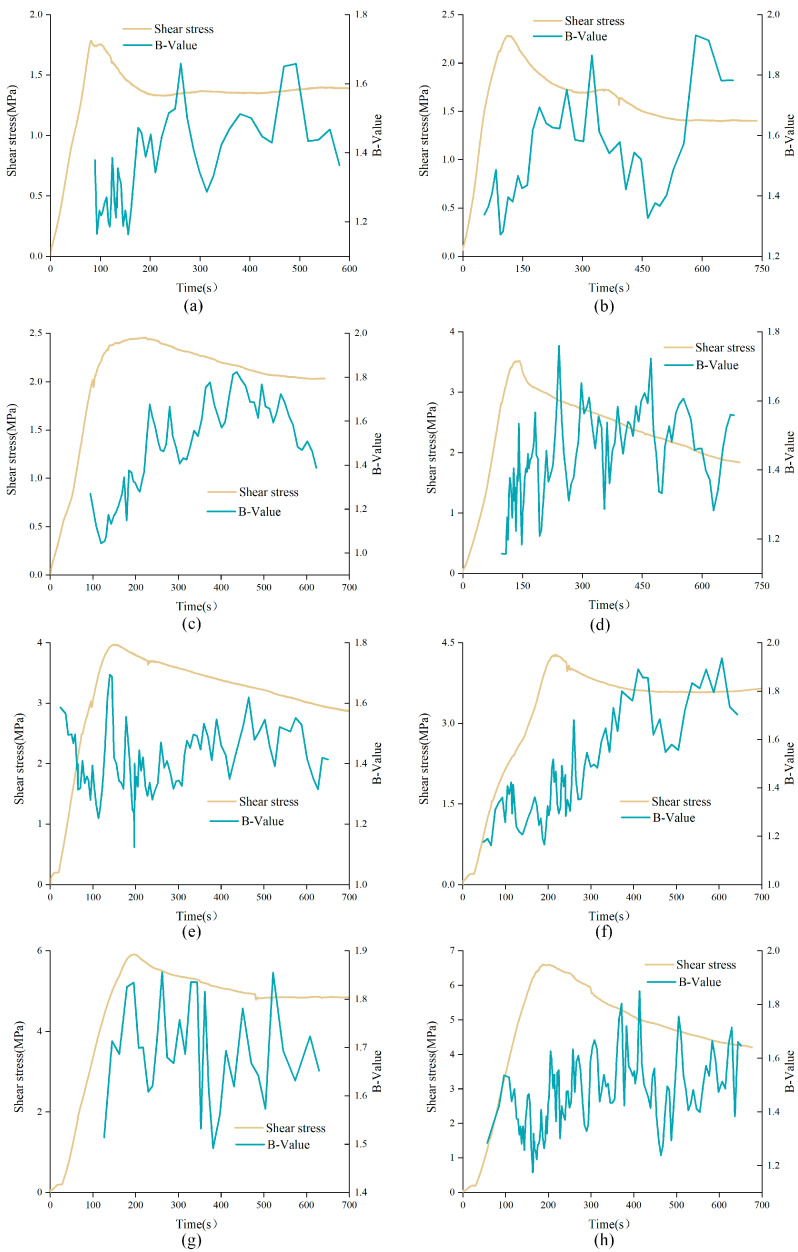
*b*-value curves of unanchored structural planes with different JRC under shearing: (**a**) DS-02-0-2, (**b**) DS-68-0-2, (**c**) DS-1214-0-2, (**d**) DS-1820-0-2, (**e**) DS-02-0-5, (**f**) DS-68-0-5, (**g**) DS-1214-0-5, and (**h**) DS-1820-0-5 (specimen ID: DS-ab-α-σ, where DS represents direct shear experiment, ab represents the range of JRC, α means the anchorage angle, and σ means the normal stress).

The variations of *b*-value with time in direct shear test of unanchored structural planes with different JRC under low normal stress (2 MPa) and high normal stress (5 MPa) were analyzed, as shown in Figure 12. As can be seen from the figure, the *b*-value fluctuated between the interval [1.0, 2.0] during the whole shear experiment, presenting an overall upward trend. In addition, from the overall trend, the *b*-value curve showed a significant correlation with shear stress curve, which can be roughly divided into three phases. The first phase corresponds to pre-peak stage. In this phase, the *b*-value curve showed a small wavelike rise and was relatively dense. The *b*-value was in a relatively low range, indicating that the failure of the specimen in this phase was mainly caused by progressive microcrack initiation. In the second phase, corresponding to the strain-softening stage, it can be found that the *b*-value curve underwent a large change in a short time near the peak strength point, which means that the structural plane had a sudden failure in this stage, and the shear stress curve also showed a rapid drop during this period. It is worth noting that for unanchored structural plane with JRC of 0–2 or 12–14, *b*-value corresponding to the peak shear strength tended to be at a high level, and the *b*-value curve climbed before the peak and fell after the peak. However, for unanchored structural plane with JRC of 6–8 or 18–20, *b*-value corresponding to the peak shear strength tended to be at a low level, and the *b*-value curve fell before the peak and climbed after the peak. This may due to the different failure characteristics of structural planes. The third phase corresponds to the residual stage. On the whole, the *b*-value curve tended to be sparse in this phase. This is because the structural plane was destroyed in the previous loading process. In the residual stage, only a few small convexes were cut off, and the AE phenomenon tended to be weak. 

**Figure 13:**
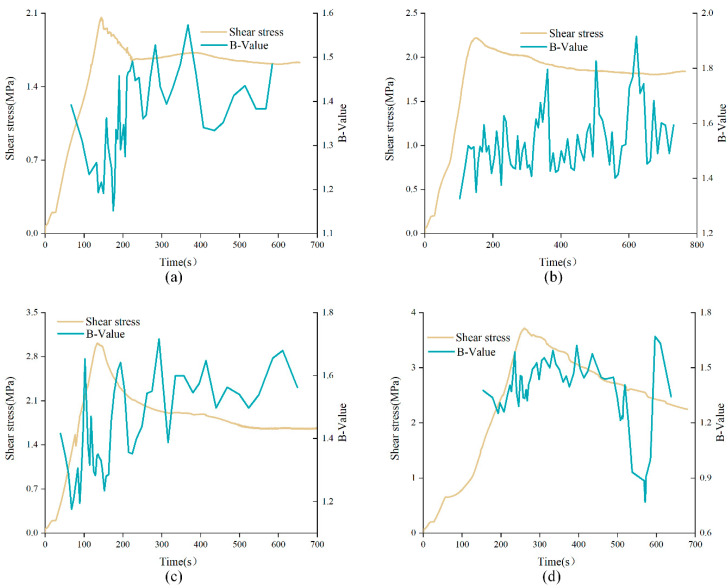
*b*-value curves of anchored structural planes with different JRC under shearing: (**a**) DS-02-60-2, (**b**) DS-68-60-2, (**c**) DS-1214-60-2, and (**d**) DS-1820-60-2 (specimen ID: DS-ab-α-σ, where DS represents direct shear experiment, ab represents the range of JRC, α means the anchorage angle, and σ means the normal stress).

From the above, the optimal angle range of bolt is [45°, 60°] Therefore, *b*-value curves of anchored structural planes with α of 60° were selected for analysis, as shown in Figure 13. Similar to the *b*-value evolution curves of unanchored structural plane, *b*-value curves of anchored structural planes were relatively dense in the initial loading stage, and tended to be sparse in the residual stage, presenting an overall upward trend. However, the *b*-value of anchored structural plane around the peak of shear stress curve was at a lower position, regardless of the variation of JRC. In the initial shear stage, that is, the elastic deformation stage, *b*-value of anchored structural plane with JRC of 0–2 and 12–14 decreased sharply, while the *b*-value of the anchored structural plane with JRC was 6–8 and 18–20 fluctuated slightly. This is mainly due to the fact that the anchored structural plane with JRC of 0–2 and 12–14 had fewer bulges, and crack propagation is relatively faster under shearing. 

**Figure 14:**
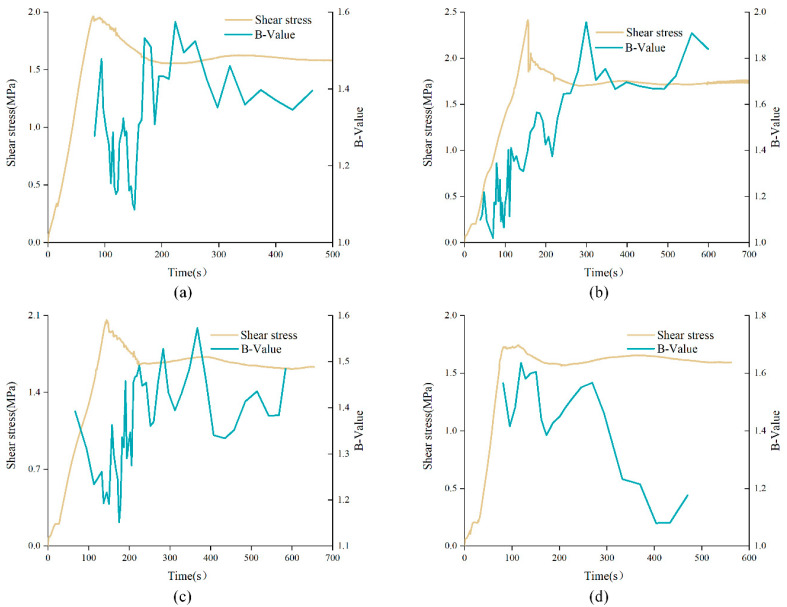
*b*-value curves of anchored structural planes with different anchorage angles under shearing: (**a**) DS-02-30-2, (**b**) DS-02-45-2, (**c**) DS-02-60-2, and (**d**) DS-02-90-2 (specimen ID: DS-ab-α-σ, where DS represents direct shear experiment, ab represents the range of JRC, α means the anchorage angle, and σ means the normal stress).

It can be found that in the shear test that when the anchorage angle was 30° or 45°, the bolt usually did not have obvious deformation, but when the anchorage angles were 60° or 90°, the bolt showed obvious deformation. Anchored structural planes with JRC of 0–2 subjected to 2 MPa normal stress were used to analyzed the influence of anchorage angle variation on characteristics of *b*-value curves, as shown in Figure 14. All curves were relatively dense in the initial loading stage, and tended to be sparse in the residual stage, indicating that AE events were few in the residual stage. In addition, there were obvious differences in *b*-value curves of anchored structural planes with different anchorage angles. In the pre-peak stage, the *b*-value curve showed an overall upward trend for anchored structural plane with anchorage angles of 30° or 45°, while for anchored structural planes with anchorage angle of 60° or 90°, the *b*-value presented an overall downward trend. The variation of *b*-value for the anchored structural plane with anchorage angle of 30° or 45° was small. However, for the *b*-value curve of the anchored structural plane with anchorage angle of 60° or 90°, there existed an obvious drop in the residual stage. This may be due to the obvious deformation of the bolt in the shear process, which will squeeze the rock mass around the bolt and cause a large fracture zone.

### 4.3. Analysis of RA-AF

In the process of rock deformation, the micro-fracture modes are mainly tensile failure and shear failure, and AE characteristics induced by these two fracture modes are different. In the study of AE characteristics, characteristic parameter analysis is a common method for signal processing. Among them, RA and AF derived from AE timing parameters are often used to characterize the generation mechanism of AE sources. RA and AF are defined as:

RA = Rise time/Amplitude(7)

(8) AF=Ring count/Duration

The units of RA and AF are ms/V and kHz, respectively.

Based on the previous studies [46,47,48], AE signal characteristics of shear cracks are high RA value and low AF value, and AE signal characteristics of tensile cracks are high AF value and low RA value.

**Figure 15:**
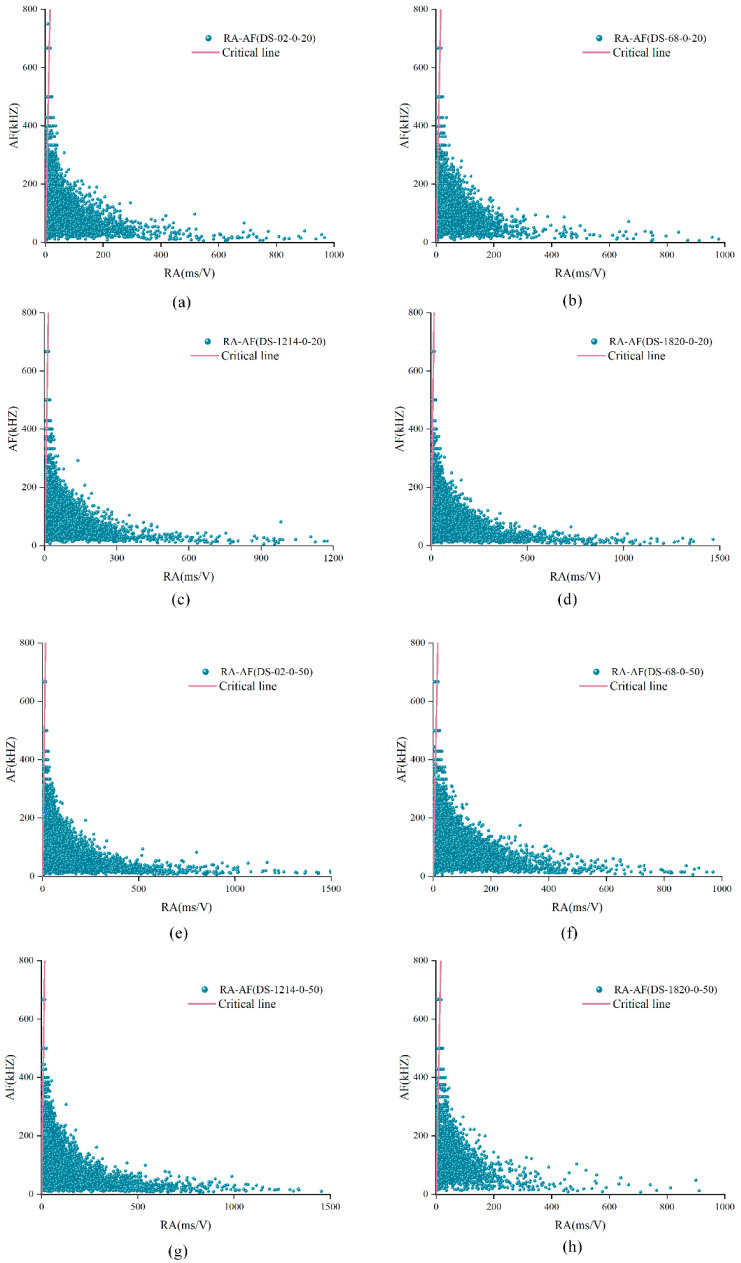
RA-AF distribution of unanchored structural planes. (**a**) DS-02-0-2 (**b**) DS-68-0-2 (**c**) DS-1214-0-2 (**d**) DS-1820-0-2 (**e**) DS-02-0-5 (**f**) DS-68-0-5 (**g**) DS-1214-0-5 (**h**) DS-1820-0-5.

As the normal stress increases, the joint’s asperity degradation degree of structural planes increases. Therefore, adopting the RA-AF distribution of unanchored structural planes under the normal stress of 2 MPa and 5 MPa were analyzed, as shown in Figure 15. It can be seen from the figure that the distribution of RA-AF was roughly a triangle, and most of the signals were distributed under the red dotted line, indicating that shear cracks occupied a dominant position in the experimental process. AF values were mostly distributed in the range of 0–400, and there were few shear cracks with RA values greater than 800 and tensile cracks with AF values greater than 600.

#### 4.3.1. Variation of Cumulative Proportion of Shear-Tensile Cracks

**Figure 16:**
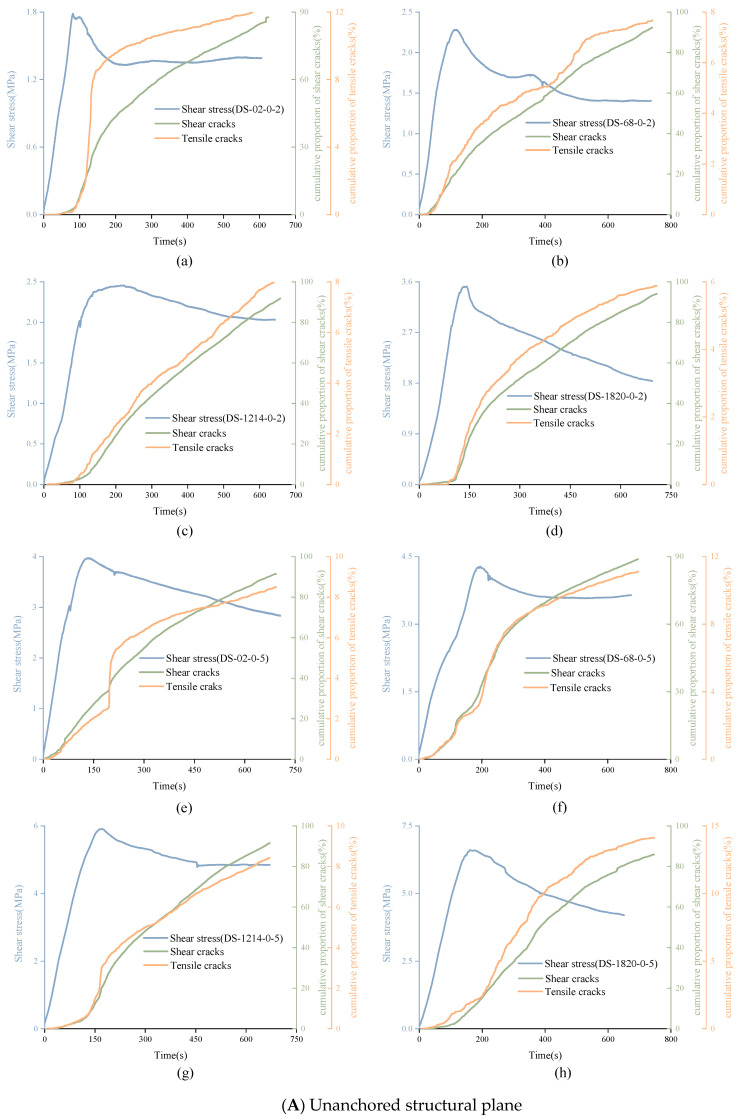

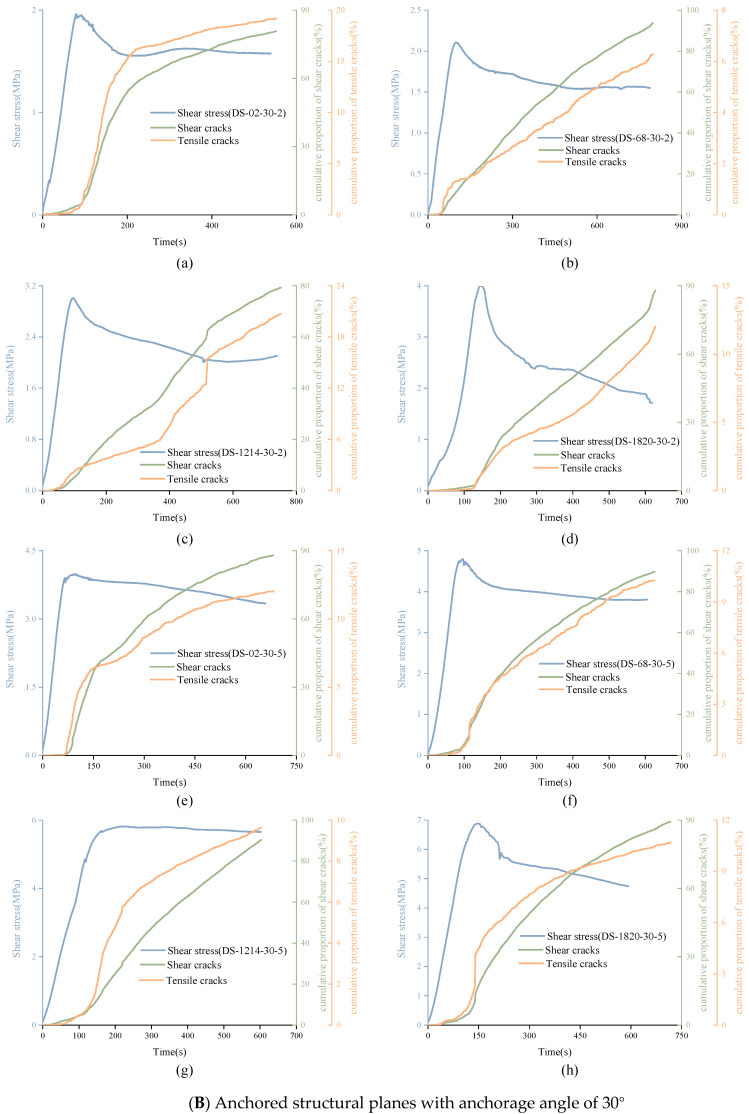

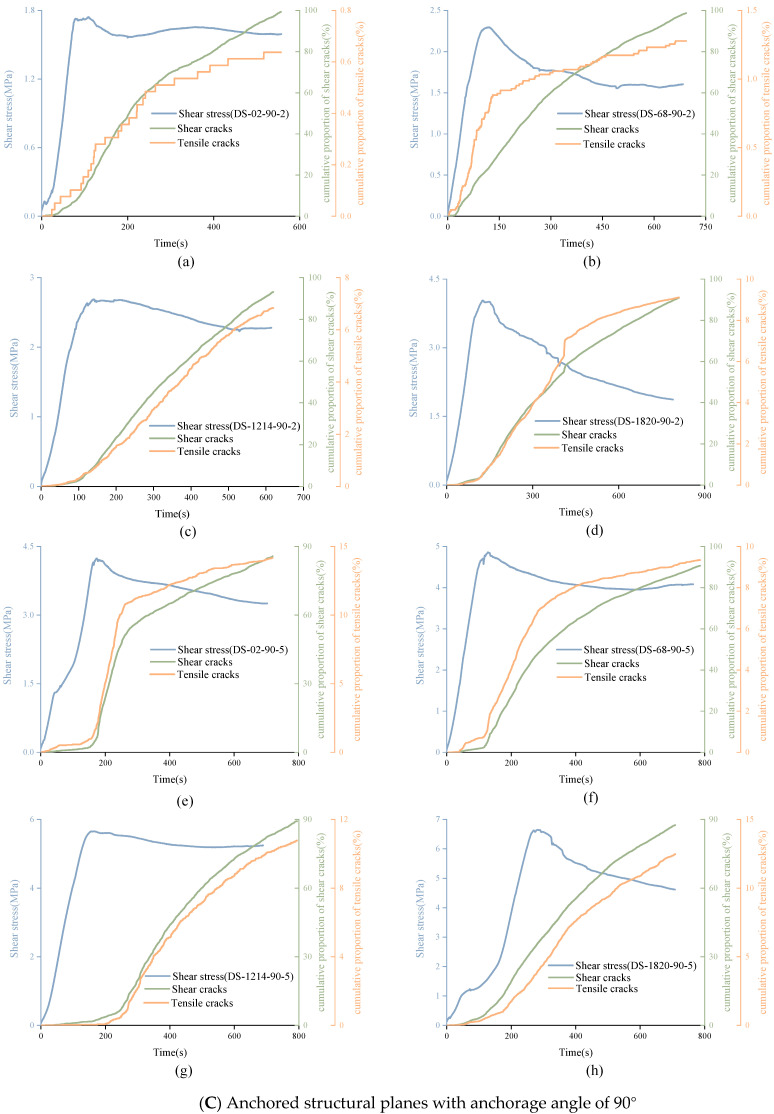
Variation trend of cumulative proportion of shear cracks and tensile cracks with time. ((**A**) (**a**) DS-02-0-2 (**b**) DS-68-0-2 (**c**) DS-1214-0-2 (**d**) DS-1820-0-2 (**e**) DS-02-0-5 (**f**) DS-68-0-5 (**g**) DS-1214-0-5 (**h**) DS-1820-0-5); ((**B**) (**a**) DS-02-30-2 (**b**) DS-68-30-2 (**c**) DS-1214-30-2 (**d**) DS-1820-30-2 (**e**) DS-02-30-5 (**f**) DS-68-30-5 (**g**) DS-1214-30-5 (**h**) DS-1820-30-5); ((**C**) (**a**) DS-02-90-2 (**b**) DS-68-90-2 (**c**) DS-1214-90-2 (**d**) DS-1820-90-2 (**e**) DS-02-90-5 (**f**) DS-68-90-5 (**g**) DS-1214-90-5 (**h**) DS-1820-90-5).

By calculating and accumulating the proportion of shear cracks and tensile cracks corresponding to each time point, the variation trend of the cumulative proportion of shear cracks and tensile cracks can be obtained, as shown in Figure 16. Through comparison, it can be found that the proportion of shear cracks was much higher than that of tensile cracks, accounting for more than 85%. This means that the tangential force was the main reason for the failure of structural plane in the shear experiment. For the unanchored structural plane with JRC of 0–2, the cumulative proportion curve of tensile cracks showed an obvious three-stage pattern, which has a significant correlation with the shear stress curve. In the pre-peak stage, the curve rose slowly. When the shear stress reached the peak, the tensile signal curve rose rapidly and the upward trend of tensile cracks curve slowed down after the shear stress entering the residual stage. The rising trend of the cumulative proportion curve of shear cracks remained almost unchanged during the whole loading process except that it was relatively flat at the initial stage of loading. It was found that the variation trend of shear and tensile cracks accumulation curves of the anchored structural plane with JRC of 0–2 was consistent, which are all three-stage characteristics. In addition, for unanchored structural planes with JRC of 6–8 and 18–20, the cumulative proportion curves of shear cracks and tensile cracks were three-stage patterns, but the curve was coarser. This mainly because the structural planes with JRC of 6–8 and 18–20 are composed of many small bulges, which were cut off in the experiment, resulting in fluctuation. For the anchored structural plane with JRC of 6–8 and 18–20, the existence of the bolt makes the failure characteristics of structural plane more complex, which leads to more complicated characteristics of the signal accumulation curve. Generally speaking, the cumulative proportion curve is easier at showing a three-stage type when the normal stress is high. For the structural plane with JRC of 12–14, whether anchored or not, the signal accumulation curve will show three-stage type when the normal stress equals 5 MPa.

#### 4.3.2. Variation of Real-Time Proportion of Shear Cracks

**Figure 17:**
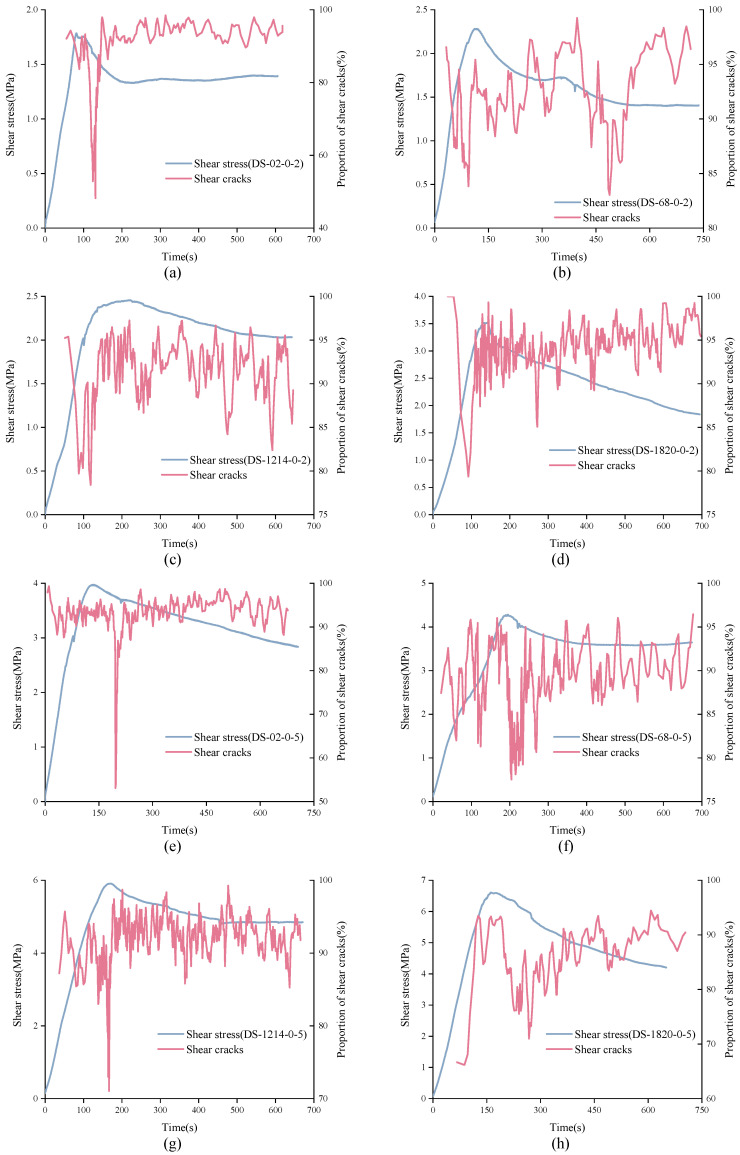
Variation trend of real-time shear cracks’ proportion curves of unanchored structural planes. (**a**) Ds-02-0-2 (**b**) Ds-68-0-2 (**c**) Ds-1214-0-2 (**d**) Ds-1820-0-2 (**e**) Ds-02-0-5 (**f**) Ds-68-0-5 (**g**) Ds-1214-0-5 (**h**) Ds-1820-0-5.

**Figure 18:**
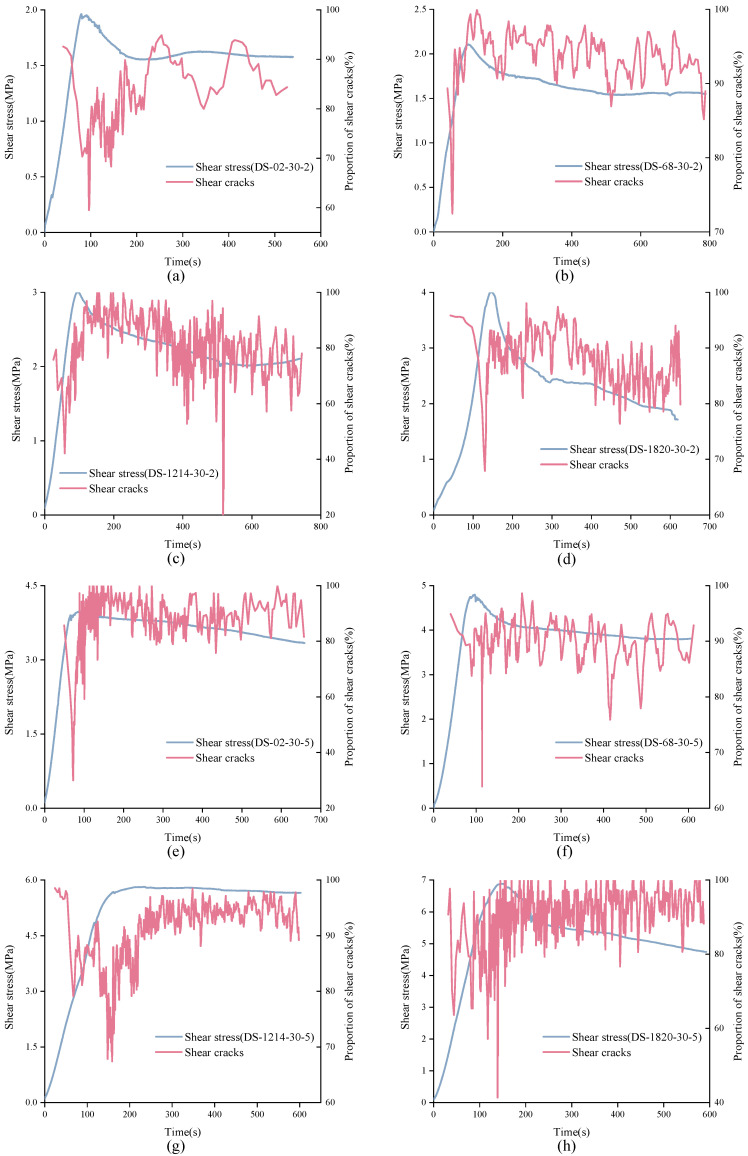
Variation trend of real-time shear cracks’ proportion of anchored structural planes with anchorage angle of 30°. (**a**) DS-02-30-2 (**b**) DS-68-30-2 (**c**) DS-1214-30-2 (**d**) DS-1820-30-2 (**e**) DS-02-30-5 (**f**) DS-68-30-5 (**g**) DS-1214-30-5 (**h**) DS-1820-30-5.

**Figure 19:**
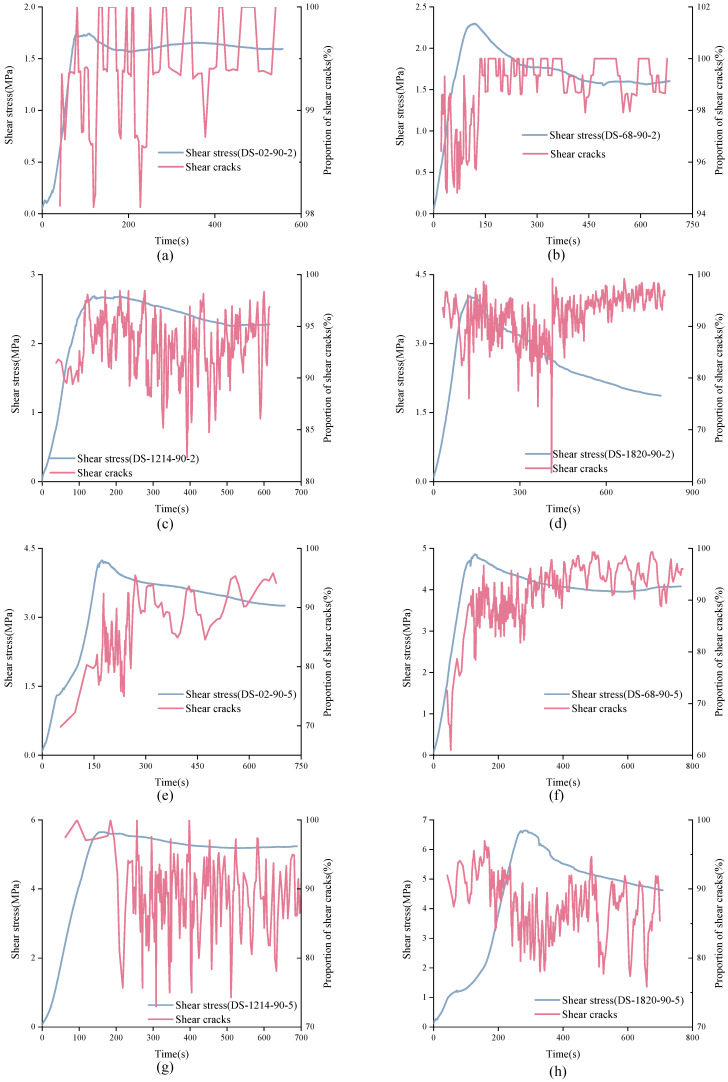
Variation trend of real-time shear cracks’ proportion curves of anchored structural planes with anchorage angle of 90°. (**a**) DS-02-90-2 (**b**) DS-68-90-2 (**c**) DS-1214-90-2 (**d**) DS-1618-90-2 (**e**) DS-02-90-5 (**f**) DS-68-90-5 (**g**) DS-1214-90-5 (**h**) DS-1820-90-5.

It is not difficult to see from Figure 16 that in the direct shear test, the cumulative proportion of shear cracks was much larger than that of tensile cracks. Generally, the cumulative proportion of tensile cracks was around 10% or less. However, from the trend of cumulative proportion curve, the real-time proportion of shear cracks and constant signal was not constant. Understanding the real-time change of shear cracks and tensile cracks is of great significance to study the failure process of structural plane. Considering that there are too many AE signals, it is difficult to find the law if calculating the signal type corresponding to each AE signal. Thus, the real-time change trend of shear cracks can be approximately reflected by calculating the proportion of shear cracks in every 200 data, as shown in Figure 17, Figure 18 and Figure 19. 

In Figure 17, the characteristics of real-time shear crack curves of structural planes with different JRC are different. For unanchored structural planes with JRC of 0–2 and 12–14, the real-time proportion of shear cracks showed the same variation trend. At the initial stage of loading, shear cracks accounted for a large proportion of about 0.9. As loading continues, the real-time proportion curve of shear cracks showed a sudden drop and then recovered to a high level in a short time. The difference is that the shear cracks’ real-time proportion curve of structural plane with JRC of 0–2 suddenly dropped in the strain-softening stage, while that of the structural plane with JRC of 12–14 dropped in the pre-peak stage. Besides, for unanchored structural planes with JRC of 0–2, the real-time ratio of shear cracks could be as low as 50%. For structural planes with JRC of 6–8, the proportion of shear cracks was always at a high level, fluctuating between 0.8 and 1.0. For structural planes with JRC of 18–20, the normal stress affected the characteristics of shear cracks’ real-time proportion curve. Under low normal stress, the curve decreased first and then rose, and finally rose slowly in fluctuation. The variation characteristics of shear cracks real-time proportion curve of structural plane under high normal stress rose first, then fell, and finally rose. It is worth noting that although the proportion of shear cracks fluctuated greatly over time, the proportion of shear cracks was always more than 50%. Figure 18 shows the variation process of shear cracks’ real-time proportion for anchored structural planes with anchorage angle of 30°. For structural planes at this anchorage angle, the real-time shear crack proportion curves had the same variation characteristics. The real-time ratio of shear cracks almost remained above 80%, but there was a sudden drop, and sometimes even lower than 30%. Figure 19 shows the variation characteristics of shear cracks real-time proportion for anchored structural planes with anchorage angle of 90°. The variation trend of shear cracks proportion for anchored structural planes with JRC of 0–2 and 6–8 was consistent under low normal stress and high normal stress, and the proportion of shear cracks in the residual stage was higher than that in the pre-peak stage and strain-softening stage. The real-time shear cracks’ proportion curve of the anchored structural plane showed a gentle overall change trend, but fluctuated greatly in a small area. For anchored structural planes with JRC of 18–20, the real-time shear cracks’ proportion curve decreased first and then increased.

## 5. Conclusions

In this study, direct shear tests were carried out on anchored structural planes with different JRC, AE technology was used to explore the AE characteristics of structural planes during shearing, and the following conclusions are drawn:

(1) The larger the normal stress, the larger the peak shear strength of the anchored structural plane. Under the same normal stress, compared with the peak shear strength, the residual strength of structural planes with JRC of 6–8 and 18–20 decreased more, and that of structural planes with JRC of 0–2 and 12–14 decreased less. The peak shear strength of the anchored structural plane increased and then decreased with the variation of anchorage angle, and always reached the maximum value at 45° or 60°, which means the optimal installation angle of the bolt is in the range of [45°, 60°].
(2) According to the AE monitoring results, the ring-down count rises first, then decreases and finally flattens, showing an obvious correlation with the shear stress curve. The ring-down count still kept a certain degree in the residual stage, which was about 60. The cumulative ring-down count curve was characterized by three-stage and the increase of normal stress accelerated the curve entering the rapid growth stage. The *b*-value curve was dense at the initial loading stage and tended to be sparse in the residual stage. Its variation trend mainly depended on the topography of structural plane, not only affected by the value of JRC. The influence of anchorage angle on *b*-value variation characteristics mainly depended on whether the bolt would be deformed during shearing.
(3) Through AE experiments, the cumulative ratio of shear cracks could reach 85%, which is much higher than that of tensile cracks. The cumulative proportion curve of tensile cracks showed a three-stage pattern and the correlation with the shear stress curve was more significant. Besides, the higher the normal stress, the easier the signal cumulative proportion curve appearing in three-stage form. The proportion of shear cracks and tensile cracks in the experiment changed dynamically. For unanchored structural planes, the proportion of shear cracks was more than 50% in the whole experiment. For anchored structural planes, the proportion of tensile cracks may exceed that of shear cracks, sometimes even up to 80%.

In this paper, AE technology was used to study the micro-failure characteristics of anchored structural with different JRC. However, this paper considers only the standard JRC profiles, which are generated from 2D profiles. Natural rock joints in nature are usually random, three-dimensional and imperfectly matched. However, it is difficult to prepare specimens with imperfectly matched structural planes through the fabrication method in this paper. The research on the mechanical properties of anchored natural structural planes can be carried out in the future.

## Figures and Tables

**Table 1:** Mechanical parameters of cement mortar.

Mechanical Parameters	Value
Unconfined compression strength (MPa)	34.13
Elastic modulus (GPa)	5.27
Tensile strength (MPa)	2.98
Cohesion (MPa)	13.79
Friction angle (°)	27.92
Poisson’s ratio	0.21

**Table 2:** Shear strength parameters of unanchored specimens with different JRC.

Parameters	JRC = 0–2	JRC = 6–8	JRC = 12–14	JRC = 18–20
Cohesion-like stress (Mpa)	0.34	0.82	0.14	1.73
Friction angle (°)	36.2	34.0	47.7	44.2

## Data Availability

The data used to support the findings of this study are available from the corresponding author upon request.
